# Effects of remote ischemic conditioning on microcirculatory alterations in patients with sepsis: a single-arm clinical trial

**DOI:** 10.1186/s13613-021-00848-y

**Published:** 2021-04-07

**Authors:** Inga Kiudulaite, Egle Belousoviene, Astra Vitkauskiene, Andrius Pranskunas

**Affiliations:** 1 Department of Intensive Care Medicine, Lithuanian University of Health Sciences, Eiveniu str. 2, 50161 Kaunas, Lithuania; 2 Department of Laboratory Medicine, Lithuanian University of Health Sciences, Eiveniu str. 2, 50009 Kaunas, Lithuania

**Keywords:** Microcirculation, IDF imaging, Remote ischemic conditioning

## Abstract

**Background:**

Remote ischemic conditioning (RIC) is a promising technique that may protect organs and tissues from the effects of additional ischemic episodes. However, the therapeutic efficacy of RIC in humans with sepsis remains unknown. We hypothesized that RIC might improve sublingual microcirculation in patients with sepsis.

**Methods:**

This prospective single-arm trial was performed in a mixed ICU at a tertiary teaching hospital. We included patients with sepsis or septic shock within 24 h of ICU admission. The RIC procedure comprised 3 cycles of brachial cuff inflation to 200 mmHg for 5 min followed by deflation to 0 mmHg for another 5 min. The procedure took 30 min. RIC was performed at the time of study inclusion and repeated after 12 and 24 h. Sublingual microcirculatory measurements were obtained before and after each RIC procedure using a Cytocam^®^-incident dark-field (IDF) device (Braedius Medical, Huizen, The Netherlands). The microcirculatory data were compared with a historical control. Data are reported as the medians along with the 25th and 75th percentiles.

**Results:**

Twenty-six septic patients with a median age of 65 (57–81) years were enrolled in this study. The median Acute Physiology and Chronic Health Evaluation (APACHE) II and Sequential Organ Failure Assessment (SOFA) scores at admission were 20 (13–23) and 10 (9–12), respectively. All patients were receiving vasopressors. After the 1st RIC procedure, the microvascular flow index (MFI) and the proportion of perfused vessels (PPV) among small vessels were significantly higher than before the procedure, with pre- and post-treatment values of 2.17 (1.81–2.69) and 2.59 (2.21–2.83), respectively, for MFI (*p* = 0.003) and 87.9 (82.4–93.8) and 92.5 (87.9–96.1) %, respectively, for PPV (*p* = 0.026). This result was confirmed by comparison with a historical control group. We found no change in microcirculatory flow or density parameters during repeated RIC after 12 h and 24 h.

**Conclusion:**

In patients with sepsis, the first remote ischemic conditioning procedure improved microcirculatory flow, whereas later procedures did not affect sublingual microcirculation.

*Trial registration* NCT04644926, http://www.clinicaltrials.gov. Date of registration: 25 November 2020. Retrospectively registered, https://clinicaltrials.gov/ct2/show/NCT04644926.

## Background

The global mortality rate from sepsis is considerably high [1]. Sepsis is an infection complicated by impaired microcirculation and abnormal inflammatory response, which eventually leads to multiple organ damage. Thus, altered microcirculation is the cornerstone of sepsis [2]. The severity of microcirculatory alterations during sepsis is related to mortality [3]. Therefore, new treatment options for improving or preventing microcirculatory disturbances during sepsis need to be developed. For practical reasons, studies in critically ill patients have usually examined sublingual microcirculation using sidestream dark-field (SDF) or incident dark-field (IDF) imaging.

Remote ischemic conditioning is a novel treatment modality in which tissues are subjected to short cycles of ischemia followed by reperfusion, resulting in a reduction of ischemia–reperfusion injury at a remotely injured site, such as the heart, brain, lungs or intestine. The most commonly employed technique is 3 to 4 cycles of inflation for 5 min followed by deflation for 5 min using a standard blood pressure cuff on the upper arm or thigh [4]. There are 3 variants of remote conditioning interventions based on the timing of intervention in relation to the ischemic insult and reperfusion: remote ischemic preconditioning, remote ischemic conditioning (RIC, initiated at the time of ischemia), and remote ischemic postconditioning (initiated at the reperfusion stage). All these variants may have clinical benefits for multiple organs at the same time [4, 5]. Clinical studies have shown that RIC may improve outcomes in patients with acute ST-segment elevation myocardial infarction [6] and acute ischemic stroke [7]. Although the exact mechanisms of RIC remain uncertain, it is thought that the conditioning stimulus is transferred through both humoral and neural pathways [8]. The endogenous protection provided by RIC is partially attributed to reduction of the immunoinflammatory response and oxidative stress [9–11]. RIC has the potential to improve endothelial function [12], increase red blood cell (RBC) deformability [13] and reduce leukocyte adhesion [14]. The latter effects of RIC might be responsible for improving microcirculation. A study showed that remote ischemic preconditioning in combination with conditioning improved hemodynamics, preserved microcirculation, and led to an increased survival rate in sheep with sepsis [15]. However, the therapeutic efficacy of RIC in sepsis, which is a disease of the microcirculation initiated by early microcirculatory dysfunction, remains unknown in humans. We hypothesized that RIC might improve sublingual microcirculation in sepsis.

## Methods

### Patients

This single-center, single-arm, open-label clinical trial was performed to investigate whether RIC improves sublingual microcirculation in patients with sepsis. The trial was conducted in an 18-bed mixed ICU at a tertiary teaching hospital (The Hospital of the Lithuanian University of Health Sciences). The study was approved by the Kaunas Regional Biomedical Research Ethics Committee (No. BE-2-78) and performed in compliance with the Declaration of Helsinki. Written informed consent was obtained from the patients or their next of kin, consistent with the applicable laws. We registered this trial retrospectively with ClinicalTrials.gov under the identifier NCT04644926.

Patients with sepsis or septic shock were enrolled within 24 h of ICU admission. Sepsis was defined by the Sepsis-3 criteria published by the Sepsis Redefinitions Task Force [16]. The exclusion criteria were age < 18 years, pregnancy, advanced malignancy, peripheral artery disease affecting both arms, oral mucosal inflammation or injury, and technical difficulties in obtaining sublingual images.

### Study protocol

Once the participants were enrolled in the study, demographic variables; routine laboratory test results; arterial and central venous blood gas measurements; systemic hemodynamics; ventilator settings; and other physiological parameters, including Acute Physiology and Chronic Health Evaluation (APACHE) II score and Sequential Organ Failure Assessment (SOFA) score, were obtained. The RIC intervention comprised 3 cycles of brachial cuff inflation to 200 mmHg for 5 min followed by deflation to 0 mmHg another 5 min. The procedure took 30 min. An appropriate brachial cuff was chosen according to the forearm circumference. Except for the RIC procedure, all the patients received standard sepsis treatment as per international guidelines. RIC intervention was provided by an investigator who was not involved in patient treatment. The RIC procedure is considered safe. To the best of our knowledge, there have not been any procedure-related adverse events in previous clinical trials on non-septic patients. The RIC procedure was performed at inclusion and repeated after 12 h and 24 h. Evaluation of sublingual microcirculation, systemic hemodynamics, and arterial and central venous blood sampling was performed before and after each RIC intervention. Systemic hemodynamic parameters were measured using a femoral artery thermodilution catheter in combination with a central venous line (PiCCO, Pulsion Medical Systems, Munich, Germany). In addition, blood samples for assessing the inflammatory mediator IL-6 were collected at inclusion and after 24 h. The blood samples obtained were immediately centrifuged and stored at − 80 °C for further analysis.

We compared this intervention with standard care using a historical control group of patients with sepsis treated in the same ICU.

### Evaluation of the microcirculation

Sublingual microcirculation images were obtained using a Cytocam^®^-IDF device (Braedius Medical, Huizen, The Netherlands). This device is technically and optically optimized for visualizing microcirculation on organ surfaces. IDF imaging is based on the principle that emitted green light (wavelength 530 nm) is absorbed by hemoglobin in RBCs. Therefore, RBCs are visualized as black or gray bodies during imaging. The vessel walls are not visualized; therefore, vessels can be detected only by the presence of RBCs. A validation study showed that Cytocam-IDF imaging yielded better image quality than SDF imaging [17]. We followed the recommendations published by experts for quality and analysis of the obtained images [18]. Images from at least 3 areas were acquired and stored on a computer. Image clips were exported for analysis using validated AVA^®^ v3.0 software (Microvision Medical, Amsterdam, The Netherlands) [19]. Skilled investigators analyzed the video clips offline in a blinded manner and in random order to prevent coupling. Each image was divided into 4 equal quadrants. Flow in small vessels was classified semiquantitatively (no flow: 0; intermittent flow: 1; sluggish flow: 2; continuous flow: 3). We calculated the microvascular flow index (MFI) as the sum of each quadrant score divided by the number of quadrants in which the vessel type was visible [20]. We calculated the total vessel density (TVD) of small vessels using the AVA software package, and the cutoff diameter for small vessels (mostly capillaries) was < 20 μm. The proportion of perfused vessels (PPV) among small vessels was computed by dividing the perfused small vessel length by the total length of small vessels. The perfused vessel density (PVD) of small vessels was calculated by measuring the density of perfused small vessels within the field of view (computed as the proportion of perfused vessels multiplied by the total vessel density). The De Backer score was calculated as the number of small vessels crossing three equally spaced vertical lines and three horizontal lines in the image field divided by the total length of the lines.

### Historical control group

To avoid time-related bias, the RIC group was compared with standard care using a historical control group. The control group was formed from septic patients in a recent randomized study in the same ICU with the same inclusion criteria as in the present study. This study was conducted between January 2019 and January 2021. Historical control group patients were selected by propensity score matching method with a caliper width of 0.2 of the standard deviation of the logit of the propensity score and matched for the following criteria: sex, age, APACHE II, SOFA, time after admission to ICU, dose of vasopressors, baseline MFI and PPV of small vessels, and arterial lactate. Evaluation of sublingual microcirculation in the control group was performed using a Cytocam^®^-IDF device (Braedius Medical, Huizen, The Netherlands) at inclusion and repeated after 30 min, 12 h, and 24 h.

### Statistical analysis

The primary aim of the study was to analyze the difference between pre- and post-RIC values of MFI and PPV for small sublingual vessels in patients with sepsis. Considering the detected means and standard deviations in pilot observations, we estimated that the sample should include at least 21 patients to assess the effect of RIC on the MFI and PPV of small vessels (power 80%, alpha risk 5%). The microcirculatory data were compared with a historical control group selected by propensity score matching with the Statistical Package for Social Sciences (SPSS 27 for Windows, Chicago, IL, USA).

The distribution of quantitative variables was tested using the Kolmogorov–Smirnov normality test. Since most of the parameters showed a nonnormal distribution, data are presented as the median (interquartile range, IQR) and analyzed with non-parametric tests. Differences between pre- and post-RIC were tested using a Wilcoxon test. For the comparisons between the study patients and those from the historical control group, we used the Mann–Whitney *U* test to compare median values. The Friedman test was performed to assess the evolution of microvascular parameters at multiple time points, followed by a Wilcoxon test with Bonferroni correction for multiple pair comparisons. Correlations were tested using a Spearman correlation test. A *p* value of < 0.05 was considered significant.

## Results

The baseline characteristics of the 26 patients included in the study are presented in Table 1. The median age of the patients was 65 (57–81) years, and a majority of the patients were men (*n* = 20, 76.9%). The median time from ICU admission to inclusion in the study was 17 (12–23) h. All patients were treated with vasopressors and were intubated and mechanically ventilated. At the time of inclusion, the administered dose of norepinephrine was 0.24 (0.12–0.32) µg/kg/min, and 2 (7.7%) patients were administered a 2nd vasopressor, epinephrine. The sources of sepsis were abdominal infection (*n* = 11, 42.3%), pneumonia (*n* = 6, 23.1%), urinary tract infection (*n* = 3, 11.5%), soft tissue infection (*n* = 4, 15.4%), and other sources (*n* = 2, 7.7%). Mortality was reported in 12 patients (46.2%) in ICU.

**Table 1:** Comparison of baseline characteristics between the RIC and historical control groups

Variable	RIC group (*n* = 26)	Historical control group (*n* = 21)	*p*
Age, years	65 (57–81)	64 (53–77)	0.692
Men, *n* (%)	20 (77)	15 (71)	0.671
APACHE II	20 (13–23)	19 (14–23)	0.932
SOFA	10 (9–12)	9 (8–12)	0.104
Time after admission, h	17 (12–23)	12 (6–20)	0.159
Mortality, *n* (%)	12 (46.2)	12 (57.1)	0.459
Mean arterial pressure, mmHg	70 (62–78)	75 (65–86)	0.202
Heart rate, beats/min	109 (84–121)	108 (97–125)	0.638
Central venous pressure, mmHg	10 (7–13)	12 (10–14)	0.165
Cardiac index, *n*, L/min m^2^	15, 3.7 (2.8–4.3)	9, 3.0 (2.5–4.2)	0.503
Norepinephrine dose, *n*; µg/kg/min	26; 0.24 (0.12–0.32)	21; 0.20 (0.12–0.28)	0.377
Arterial lactate, mmol/L	1.5 (1.2–3.0)	1.7 (1.4–3.0)	0.446
PaO_2_, mmHg	102 (85–144)	86 (72–140)	0.441
Hemoglobin, g/L	102 (92–127)	110 (88–128)	0.500
CRP, mg/L	262 (151–341)	221 (133–395)	0.661

Data are presented as the median (interquartile range [IQR])

*APACHE* Acute Physiology and Chronic Health Evaluation, *SOFA* Sequential Organ Failure Assessment, *PaO*_*2*_ partial pressure of oxygen, *CRP* C-reactive protein

### Evaluation of systemic hemodynamic parameters

No significant difference in mean arterial pressure, heart rate, cardiac index, arterial lactate, or central venous saturation was observed before and after RIC at any of the 3 time points during the 24 h duration (Table 2). The dose of norepinephrine did not change significantly during the study period.

**Table 2:** Changes in systemic hemodynamic and microcirculatory parameters in septic patients before and after RIC during the study period

	I RIC at inclusion (*n* = 26)		II RIC after 12 h (*n* = 23)		III RIC after 24 h (*n* = 21)	
	Before	After	Before	After	Before	After
MAP, mmHg	70 (62–78)	74 (66–80)	72 (69–80)	73 (70–82)	70 (64–76)	71 (65–79)
HR, beats/min	109 (84–121)	108 (86–127)	112 (81–126)	106 (85–122)	101 (77–115)	92 (81–121)
CI, L/min m^2^	3.7 (2.8–4.3)	3.9 (3.0–4.2)	3.9 (3.3–5.3)	4.2 (3.5–5.0)	4.1 (3.5–5.2)	4.1 (3.6–5.1)
Lactates, mmol/L	1.4 (1.2–3.0)	1.4 (1.1–4.4)	1.3 (1.2–1.7)	1.3 (1.1–2.0)	1.4 (1.0–2.1)	1.4 (0.9–2.5)
ScvO_2_, %	77.6 (74.0–82.1)	80.4 (71.9–83.3)	81.1 (73.8–85.1)	80.4 (72.3–84.7)	73.8 (70.8–82.0)	76.3 (69.3–81.8)
TVDs, mm/mm^2^	21.5 (19.1–25.1)	21.8 (18.7–24.4)	20 (17.8–25.8)	20.6 (19.1–24.5)	22.4 (18.8–24)	22.8 (20.5–25.3)
PVDs, mm/mm^2^	18.9 (16.8–21.6)	19.6 (17.1–21.8)	18.6 (16.5–23.7)	19.3 (17.5–21.0)	19.6 (16.6–22.2)	21.5 (18.6–22.4)
PPVs, %	87.9 (82.4–93.8)	92.5 (87.9–96.1)*	91.3 (84.6–95.8)	91.5 (86.4–95.4)	90.6 (85.2–94.3)	91.4 (86.5–95.8)
MFI	2.17 (1.81–2.69)	2.59 (2.21–2.83)*	2.5 (2.00–2.83)	2.58 (2.17–2.90)	2.33 (2.17–2.67)	2.50 (2.08–2.83)
DB, n/mm	12.7 (10.7–14.2)	12.2 (10.6–13.7)	12.1 (10.2–14.7)	11.6 (10.3–14)	12.6 (11.4–13.8)	12.8 (11.6–15.1)

Data are presented as the median (interquartile range [IQR])

*MAP* mean arterial pressure, *HR* heart rate, *CI* cardiac index, *TVDs* total vascular density of small vessels, *PVDs* perfused small vessel density, *PPVs* proportion of perfused small vessels, *MFI* microcirculatory flow index, *DB* De Backer score, *ScvO*_*2*_ central venous oxygen saturation

^*^p < 0.05: significant difference before and after remote ischemic conditioning (RIC)

### Evaluation of the sublingual microcirculation

**Fig. 1:**
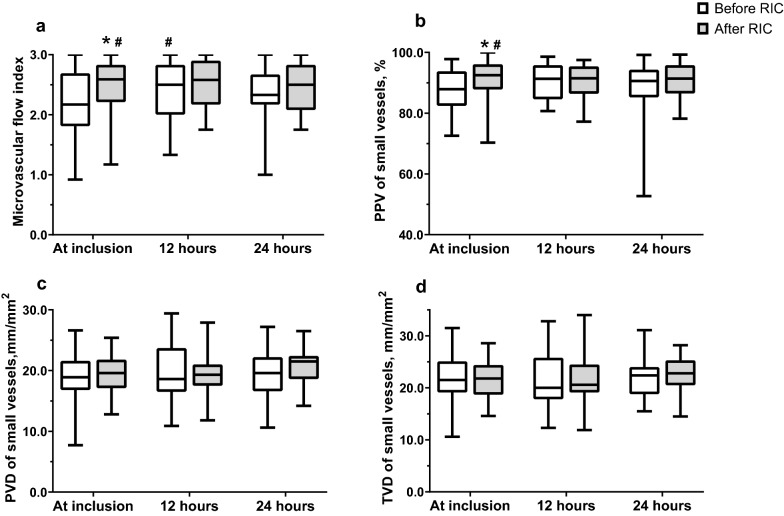
Microcirculatory parameters before and after RIC over 24 h. **a** Microvascular flow index; **b** proportion of perfused vessels (PPV) of small vessels; **c** perfused vessel density (PVD) of small vessels; **d** total vessel density (TVD) of small vessels. *p < 0.05 significant difference before and after remote ischemic conditioning (RIC). ^**#**^*p* < 0.05 compared with the historical control

After the 1st RIC, a significant increase in the MFI and PPV of small vessels was observed compared with the measurements before the procedure (2.59 (2.21–2.83) and 2.17 (1.81–2.69), *p* = 0.003; and 92.5 (87.9–96.1) % and 87.9 (82.4–93.8), *p* = 0.026, respectively). We found no change in the MFI, PPV, TVD, PVD, or De Backer scores of small vessels after repeated RIC at 12 h and 24 h (Table 2, Fig. 1).

During the 1st RIC procedure, significant correlations between the baseline MFI and PPV of small vessels and the change in MFI and PPV of small vessels (*r*_s_ = − 0.65, *p* < 0.001 and *r*_s_ = − 0.64, *p* < 0.001, respectively, Fig. 4) and between the change in MFI of small vessels and the change in cardiac index (*r*_s_ = 0.79, *p* = 0.004) were observed. However, no significant correlations were noted between changes in the MFI or PPV of small vessels and time after admission to the ICU, SOFA score, baseline norepinephrine dose, lactate, pH, changes in mean arterial pressure, heart rate, lactate, and central venous saturation (Table 3).

**Table 3:** Correlations of changes in MFI and PPV of small vessels with changes in hemodynamic parameters during the first RIC procedure

Variable	dMFIs	*p*	dPPVs	*p*
dMAP	0.08	0.698	− 0.30	0.874
dHR	− 0.20	0.326	− 0.20	0.334
dCI	0.79	0.004	0.49	0.125
dScvO_2_	0.01	0.999	− 0.10	0.626
dLactate	0.06	0.784	− 0.38	0.854

The Spearman correlation coefficient was presented. *dMFIs* changes in microvascular flow index of small vessels, *dPPVs* changes in the proportion of perfused small vessels, *dMAP* changes in mean arterial pressure, *dHR* changes in heart rate, *dCI* changes in cardiac index, *dScvO*_*2*_ changes in central venous oxygen saturation, *dLactate* changes in arterial lactate

**Fig. 2:**
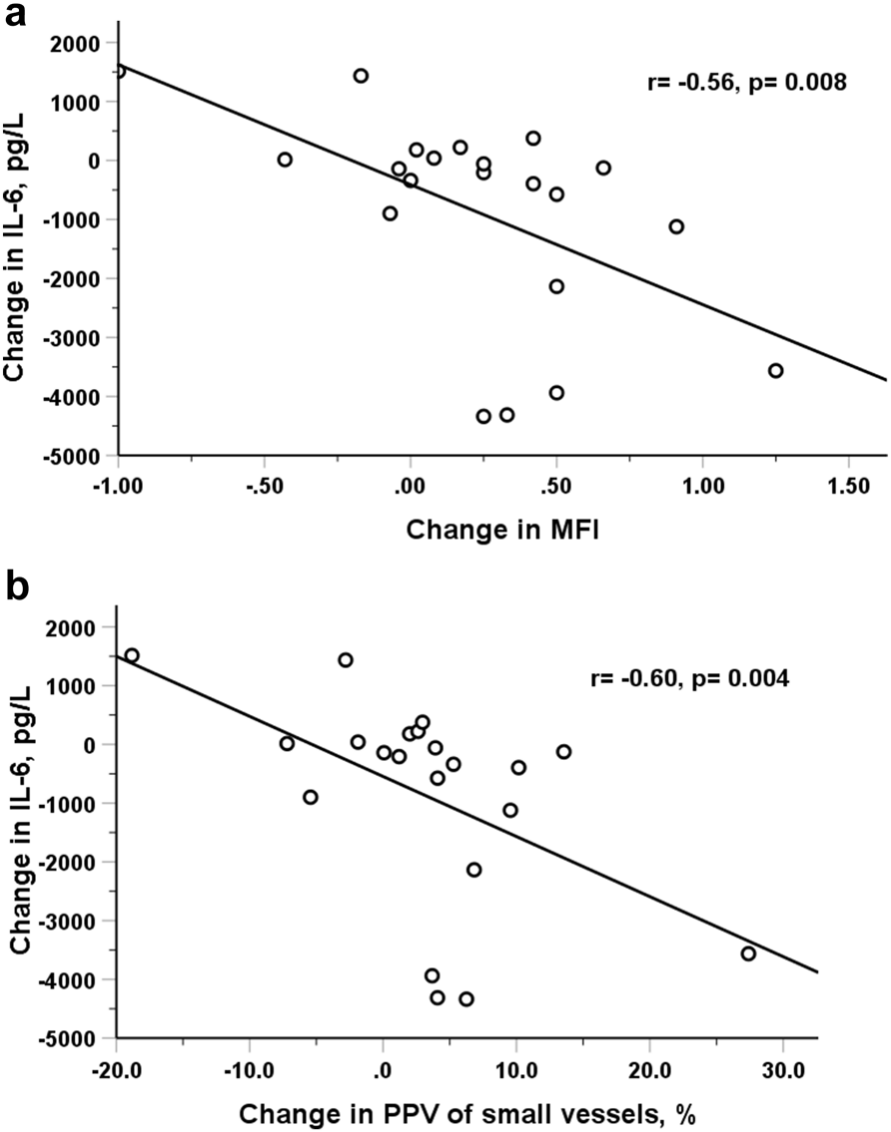
Correlation of the change in IL-6 after 24 h with **a** microvascular flow index (MFI) and **b** proportion of perfused vessels (PPV) of small vessels during the first application of RIC

A significant decrease in IL-6 concentration over 24 h (950 (424–2634) and 538 (305–1116) pg/L, *p* = 0.046) was observed. Significant correlation between the change in IL-6 levels over 24 h and the changes in MFI and PPV during the 1st RIC (*r*_s_ = − 0.56, *p* = 0.008 and *r*_s_ = − 0.60, *p* = 0.004, respectively, Fig. 2) were also observed.

#### Comparison with historical control

**Fig. 3:**
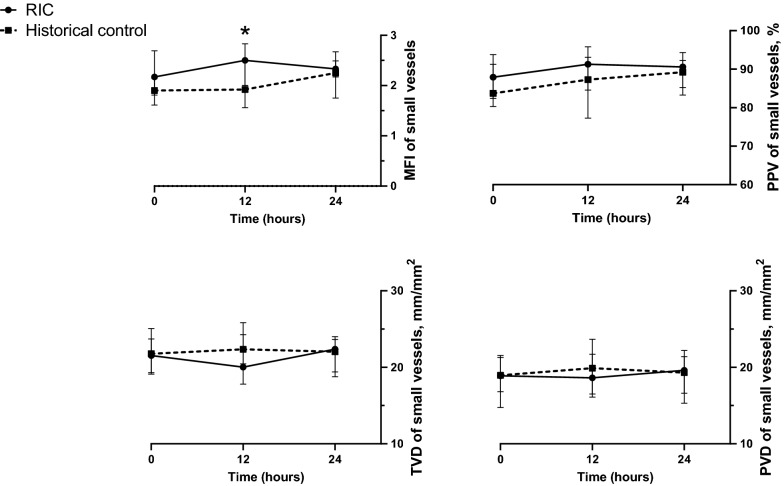
Line and scatter plot showing the median (interquartile range) of microcirculatory parameters over 24 h in the RIC and historical control groups. *MFI* microvascular flow index, *PPV* proportion of perfused vessels, *TVD* total vessel density, *PVD* perfused vessel density. *p < 0.05 compared with the historical control

The baseline characteristics of the 21 patients included in the historical control are presented in Table 1. There were no significant differences in MFI, PPV, TVD or PVD of small vessels between RIC and historical control at inclusion. In addition, we observed no change in the MFI, PPV, TVD, or PVD of small vessels over 24 h in the historical control group (Fig. 3). We detected significantly higher MFI (2.59 (2.21–2.83) vs. 2.00 (1.59–2.25), *p* < 0.001) and PPV (92.5 (87.8–96.1) vs. 85.5 (81.3–91.1) %, *p* = 0.015) of small vessels after the first RIC and significantly higher MFI at 12 h (2.50 (2.00–2.83) vs 1.92 (1.56–2.50), *p* = 0.028) in RIC patients compared to historical control (Fig. 1). We also observed a higher PPV of small vessels trend in the RIC group at 12 h compared with controls: 91.3 (84.6–95.8) vs. 87.3 (77.3–93.1) %, *p* = 0.088.

## Discussion

The major finding of the present study is that the 1st RIC improved microvascular flow in patients with sepsis, as indicated by an increase in MFI and PPV of small vessels. This result was confirmed by comparison with the historical control group. The microcirculation was unaltered by the other 2 RIC procedures after 12 and 24 h. In addition, we found a significantly higher MFI after 12 h compared to the historical control. Limited data exist on the microvascular effects of conditioning in sepsis. Cortes et al. [15] demonstrated that remote ischemic preconditioning in combination with conditioning preserved microcirculation, leading to better survival during septic fecal peritonitis in sheep. They found that the conditioned group had a higher PPV among small vessels than the control group after 6 h of sepsis, a higher MFI after 18 h, and a higher PVD of small vessels after 24 h. However, they did not evaluate changes in sublingual microcirculation before and after RIC.

The mechanism of RIC is not completely understood. RIC may act through humoral and neural pathways, thereby affecting systemic hemodynamics, endothelial function, microcirculation, and the inflammatory response. Most of the data on this procedure have been obtained from non-septic clinical or experimental studies. The phenomenon of ischemic preconditioning was first described in the canine heart, wherein brief intermittent episodes of ischemia and reperfusion of the coronary artery reduced the size of the myocardial infarct [21]. Improved coronary flow after ischemia/reperfusion was demonstrated by experimental studies on mice [22], pigs [23] and humans [24, 25]. Using intravital microscopy, Wang et al. [26, 27] demonstrated that ischemic preconditioning significantly attenuated ischemia/reperfusion‐induced vasospasm and capillary no‐reflow by increasing the average arteriolar diameter in rat skeletal muscle. Remote ischemic preconditioning stimulated nitric oxide synthase activity in RBCs and improved RBC deformability [13], reduced leukocyte adhesion in healthy volunteers [14], improved microvascular endothelial function [28] and attenuated the number of monocyte–platelet aggregates [29] in healthy volunteers. It improved microvascular endothelial function in patients with acute myocardial infarction [12, 30] and attenuated the number of monocyte–platelet aggregates in patients undergoing coronary angiography [31] and in patients undergoing ablation for atrial fibrillation [32]. Studies suggest that not only transient ischemia or interruption of blood flow but also peripheral nociception, caused by the pressure of the cuff, may trigger a protective effect on remote organs during RIC [33, 34]. The mechanisms mentioned above, mainly from preconditioning studies, could explain the positive effect of the 1st RIC on microcirculation in our study. In contrast, this effect may be intertwined with changes in systemic hemodynamics, which is indicated by a correlation between the change in MFI and the change in cardiac index during the 1st RIC.

**Fig. 4:**
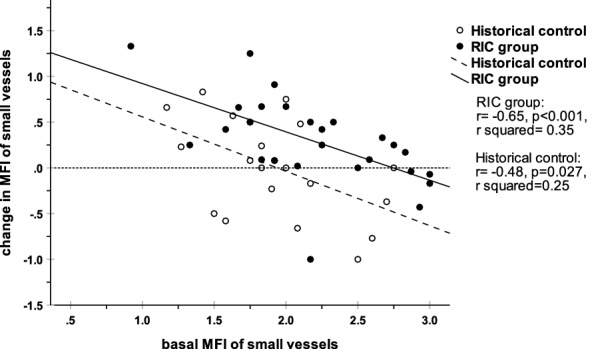
Linear regression and Pearson correlation analysis of the basal microvascular flow index (MFI) of small vessels and the change in MFI of small vessels during the first RIC period in the historical control and RIC groups

In our study, the microcirculation did not change during the other 2 RIC procedures after 12 and 24 h. However, after 12 h, we observed significantly increased MFIs and a tendency toward increased PPVs compared to historical control. This could be explained by the 2-time-window effect found in remote ischemic preconditioning studies. Studies suggested 2 time windows of protection: the 1st occurred rapidly but dissipated within 2–4 h; the 2nd occurred after 12–24 h and persisted for up to 2–3 days [5, 34, 35]. The lack of significant variation after the 2nd and 3rd RICs may simply depend upon the fact that the microvascular parameters were already at higher levels at 12 h and 24 h than at baseline. This demonstrates the regression line established during the first RIC (Fig. 4), where it can be seen that the response to RIC is markedly reduced at MFI > 2.3 and is equal to zero at MFI = 2.75.

This improvement in microcirculatory flow during the first RIC should be distinguished from the phenomenon of regression to the mean (the natural tendency of values to regress to the ‘true’ mean), especially when changes in microcirculatory flow are inversely correlated with baseline values [36]. To rule out this phenomenon or reduce its likelihood, we included in the analysis a historical control group from a recent randomized study.

We found no correlation between changes in microvascular flow and lactate concentration, vasopressor dose, patient inclusion time, or SOFA score. However, the severity and duration of sepsis may still affect the response to RIC. An experimental study with a septic mouse model found that RIC 2 h after intraperitoneal injection of lipopolysaccharide was associated with higher survival than RIC at 6 h [11]. These results suggest that the timing of RIC may be an important factor for outcomes in sepsis.

Since there is an effect on microcirculation during the first RIC and that effect may persist or reoccur at 12 h (time point of the second RIC), early application of RIC could act as a microcirculation rescue maneuver and perhaps prepare organs for new episodes of ischemia/reperfusion in the early period of sepsis treatment in the intensive care unit. The importance of early single RIC has been demonstrated in clinical trials in patients with myocardial infarction or stroke [6, 37].

We did not find a change in microvascular density, which may be related to the subsequent effects that could not be covered within this study duration. A study demonstrated that recreational marathon runners exhibited a higher functional capillary density than healthy controls [38], and this effect may be attributed to chronic ischemic (pre)conditioning.

Emerging evidence indicates that induction of endogenous protection via RIC is partially attributable to the modulation of immunoinflammatory responses. Animal studies have shown that remote ischemic conditioning [10, 11] and postconditioning [39] attenuated inflammatory responses, including IL-6 levels, and improved survival outcomes in sepsis. We also found a significant decrease in IL-6 levels after 24 h, and this change was correlated with the change in MFI and PPV during the 1st RIC.

Our study has several limitations. The first limitation of this study is its single-arm open-label design. However, the probability of change due to time or other factors is low because the study used a historical control group from a time when no increase in vasopressor doses or additional fluid infusion was applied. The historical control group was formed according to strict requirements: it met the same criteria for patient inclusion, and patients were selected by the propensity score matching method.

The most common empirically employed RIC technique in clinical trials is 3 to 4 cycles of 5 min inflation/5 min deflation using a standard blood pressure cuff on the arm or thigh [9]. In our study, the RIC procedure consisted of 3 cycles of 5 min inflation/5 min deflation using a standard blood pressure cuff on the arm. Johnsen et al. [40] compared 2, 4, 6, and 8 cycles with 2, 5, or 10 min of ischemia in each cycle in an isolated, perfused mouse heart model. They found that 4 and 6 cycles were superior to 2 cycles, while 8 cycles offered no further protection, and ischemic cycles lasting 2–5 min offered the same protection, whereas prolonged cycles lasting 10 min provided no protection. A study with healthy volunteers demonstrated that ischemic conditioning of both the upper and lower extremities can improve cutaneous blood flow, but conditioning of the upper extremity is more effective for this purpose [41]. We performed 3 repetitions of RIC in 24 h because it is not clear whether one RIC is sufficient.

## Conclusions

In patients with sepsis, the 1st RIC procedure improved microcirculatory flow, while later procedures did not affect sublingual microcirculation. Comparison with a historical control group suggests a persistent improvement in microcirculatory flow benefit after the first RIC. While its usefulness remains to be determined, RIC has the potential to serve as a clinical tool to ameliorate sepsis-induced microcirculatory alterations during the early period of sepsis treatment in the intensive care unit.

## Data Availability

The datasets used and/or analyzed during the current study are available from the corresponding author on reasonable request.

## Abbreviations

IDF: Incident dark-field illumination
SDF: Side dark field
RIC: Remote ischemic conditioning
ICU: Intensive care unit
APACHE: Acute Physiology and Chronic Health Evaluation
SOFA: Sequential Organ Failure Assessment
TVD: Total vessel density
MFI: Microvascular flow index
PPV: Proportion of perfused vessels
PVD: Perfused vessel density
HR: Heart rate
MAP: Mean arterial pressure
ScvO_2_: Central venous oxygen saturation
CRP: C-reactive protein
CI: Cardiac index
